# Differentially and Co-expressed Genes in Embryo, Germ-Line and Somatic Tissues of *Tribolium castaneum*

**DOI:** 10.1534/g3.119.400340

**Published:** 2019-05-21

**Authors:** Sher Afzal Khan, Heather Eggleston, Kevin M. Myles, Zach N. Adelman

**Affiliations:** Department of Entomology and Agrilife Research, Texas A&M University, College Station, Texas, 77843

**Keywords:** *Tribolium*, RNAseq, testis, ovary, embryo

## Abstract

Transcriptomic studies of *Tribolium castaneum* have led to significant advances in our understanding of co-regulation and differential expression of genes in development. However, previously used microarray approaches have covered only a subset of known genes. The aim of this study was to investigate gene expression patterns of beetle embryo, germ-line and somatic tissues. We identified 12,302 expressed genes and determined differentially expressed up and down-regulated genes among all samples. For example, 1624 and 3639 genes were differentially increased in expression greater than or equal to twofold change (FDR < 0.01) in testis *vs.* ovary (virgin female) and ovary *vs.* embryo (0-5 hr), respectively. Of these, many developmental, somatic and germ-line differentially expressed genes were identified. Furthermore, many maternally deposited transcripts were identified, whose expression either decreased rapidly or persisted during embryogenesis. Genes with the largest change in expression were predominantly decreased during early embryogenesis as compared to ovary or were increased in testis compared to embryo. We also identify zygotic genes induced after fertilization. The genome wide variation in transcript regulation in maternal and zygotic genes could provide additional information on how the anterior posterior axis formation is established in *Tribolium* embryos as compared to *Drosophila*. Together, our data will facilitate studies of comparative developmental biology as well as help identify candidate genes for identifying *cis*-elements to drive transgenic constructs.

The formation of independent male and female sexes is an essential and common phenomenon among animals including arthropods (West *et al.* 2002; Siebert *et al.* 2008). The majority of sexually dimorphic traits (male and female appearance and behavior) result from the differential expression of genes that are present in both sexes (Rinn and Snyder 2005). This differential gene expression is essential to initiate embryo growth, development and sex differentiation. Differences between the sexes at the genetic level can broadly be separated into two groups (1) differential gene expression, where the abundance of a specific gene transcript(s) differs between the sexes (sex-biased expression), and (2) different sex chromosomes that are present in one sex and absent in the other sex. Depending on the species, these two mechanisms can occur together; in species that lack differentiated sex chromosomes, only sex-specific gene expression patterns are observed (Pokorná and Kratochvíl 2009; Viets *et al.* 1994; Lebo *et al.* 2009; Hale *et al.* 2011). Sex bias in gene expression has been documented in multiple species including *T. castaneum* (Prince *et al.* 2010), *Drosophila* (McIntyre *et al.* 2006; Jiang and Machado 2009; Zhang *et al.* 2007) and *Caenorhabditis elegans* (Thoemke *et al.* 2005). Like other organisms, insects have shown a high amount of diversity in their sex determination mechanisms. Different insect orders use different strategies to determine their sex (Verhulst *et al.* 2010; Salz 2011; Schutt and Nothiger 2000; Matson and Zarkower 2012).

In genetic pest management programs, several methods are used or are in development for efficient sex separation of insects (Papathanos *et al.* 2009). Sex separation based solely on naturally occurring biological differences between males and females in insects has been performed but is variable (Papathanos *et al.* 2009). Some sexually dimorphic characteristics, such as body size or development rate are also influenced by natural variation, thus regular adjustment and recalibration are required for such systems to be used. For the early sterile insect technique (SIT) programs for insects, especially *Aedes aegypti*, sexes were separated using differences in pupal body size. However, many of these methods are not directly transferable to other insect species.

As an alternative, transgenic sexing systems (transgenic animals show male or female specific lethality during embryonic and early larval stages, leading to either a male or female specific population) can be developed using numerous technological approaches for the purpose of efficient sex separation in insects. Unlike biological methods for sexing, genetic methods may be more broadly applicable across species; with some minor modifications, the constructs developed for one species can be adopted to another unrelated species. For example, modifications to specific individual components of the construct such as endogenous species-specific cis elements or sex-specific splicing variants and lethal effectors may increase the efficiency of the sexing construct (to develop a construct in which a transgene leads to a high level of expression in either male or female), although simultaneously avoiding the potential negative effects of accidental species transfer. For insect transformation three components are important, (1) a gene vector (transformation method), (2) an effector gene and (3) cis elements, particularly the promoter/enhancer sequences which control transcription of the gene in a specific tissue at specific a developmental time point such as sex-specific expression of a lethal gene leading to elimination of one sex and population suppression. However, genetic sexing in a targeted species requires known differentially expressed genes in the different sexes.

Coleoptera (Beetles) are the most diverse animal group on earth and contain one fourth of all species described and includes many major pests of crop plants (Hunt *et al.* 2007). *T. castaneum* is a genetically tractable model beetle species with a published whole genome sequence (Tribolium Genome Sequencing Consortium *et al.* 2008). *Tribolium* follows the XX/XY sex determination system (Male XY and Female XX), and male and female *Tribolium* have some sexually dimorphic characters such as black spots on the first pair of legs of male adults (male beetle sexual dimorphism) which are absent in females, as well as differences in the appearance of male and female pupae (beetle pupal sexual dimorphism). To screen and separate beetles on a large scale based on these naturally occurring biological phenotypes is likely not feasible and certainly very difficult. Therefore, a study of gonadal differentiation and embryo development gene expression in *Tribolium* would be useful for the development of sexing strategies for this and other coleopterans insects important in agriculture and medicine.

Deep sequencing of mRNA (RNAseq) has been successfully used for differential gene expression analysis in a wide variety of species and conditions (Akbari *et al.* 2013; Graveley *et al.* 2011; Guo *et al.* 2018; Ruan *et al.* 2018). We performed RNAseq analysis of transcripts isolated from testis, ovary, carcasses and early embryos in *Tribolium castaneum*. We detected expression from a total of 12,302 genes in one or more of these tissues, corresponding to ∼75% of the total annotated genes (16,593) in the *Tribolium* genome. The identification of differentially or unique expressed genes reported here will facilitate future work to identify sex and early embryo specific cis elements for heterologous gene expression and to improve genetic sexing in *Tribolium* allowing for embryogenesis studies and improved pest control in the field.

## Materials and Methods

### Red beetle strain and culture

The *T. castaneum* strain was obtained from Jeff Demuth at the University of Texas at Arlington. Beetles were reared on flour medium (95% flour, 5% yeast by weight), and caged in glass jars with tight-fitting fine mesh closures. Beetles were housed in a growth chamber at 25° with 60–80% relative humidity and 12/12 hr light cycling. Populations of beetles were moved to fresh flour medium once per month with initial population densities of approximately 1−2 beetles/1 g flour medium.

### RNA isolation and RNAseq library preparation

Beetle pupae were separated based on sex prior to eclosion. Adult beetles (1–2 weeks post-eclosion) were used for embryo collection. Adult beetles were placed on flour pre-sifted with no. 50 sieve to lay eggs at 25°. The adults were removed by sifting with no. 50 sieve and transferred to fresh flour to continue embryo collection. The embryos were collected from the flour by sifting with a no. 30 sieve. Embryos were collected at 0-5 hr and 6-11 hr, respectively, in three independent biological replicates (each replicate had 80-100 embryos and were processed in a single day per sample). Similarly, ovary, testis and carcasses from male and female adult beetles at four days post eclosion (30 beetles in each replicate and processed in a single day per sample) were collected to evaluate transcriptional activity. Samples were snap frozen in liquid nitrogen and then transferred to -80° prior to RNA extraction. RNA-extraction and library preparation were done simultaneously with all samples. Total RNA from these tissues was extracted with Trizol (Invitrogen, Carlsbad, CA, USA) following the manufacturer’s instructions. After total RNA extraction, RNA was treated with Turbo DNase (Ambion/Applied Biosystems, Austin, TX). RNAseq libraries were prepared using NEBNext Poly (A) mRNA Magnetic Isolation Module (NEB #E7490) for mRNA isolation and NEBNext Ultra RNA Library Prep Kit for Illumina (*NEB #E7530S)* according to the manufacturing instructions. Each library had a peak size of approximately 300 base pairs. cDNA library quality was assessed on an Agilent 2200 TapeStation using a High Sensitivity D1000 tape. Libraries were quantified using the Invitrogen Qubit 2.0 Fluorometer High Sensitivity dsDNA assay. All libraries were normalized to a 4 nM concentration and then pooled in equal volumes for denaturing and diluting for sequencing. The library pool was denatured and diluted to a final concentration of 1.8 pM following the Illumina NextSeq Denature and Dilution protocol including a 1% PhiX DNA spike in. Sequencing was performed on an Illumina NextSeq 500, 75 cycles, high-output sequencing run generating approximately 550 Million sequencing reads. We used Illumina genome sequencing platform (NextSeq500 illumina^R^) at the Texas A&M Institute for Genome Sciences and Society Shared Molecular Genomics Core to sequence the aforementioned samples, producing unpaired 75-nt raw reads.

### Data Analysis

Sequencing bcl files were automatically uploaded to Illumina’s cloud service, BaseSpace. BaseSpace automatically generates FASTQ files, demultiplexes and trims adapter sequences. FastQC (version 0.11.5) tool was used to check the quality of the reads in each sample. HiSat2 software version 2.0.5 (Kim *et al.* 2015) was used to align reads to the *Tribolium* genome (GCA_000002335.3_Tcas5.2_genomic_RefSeqIDs.gff, obtained from www.ncbi.nlm.nih.gov) using default parameters. BEDTools (version 2.27.0) was used to produce raw counts. We used two approaches to determine which genes encode maternal, zygotic, soma or germ-line unique or increased gene expression. First, we identified genes whose expression is co-regulated across samples by using soft clustering (50% mem Soft Cluster graph) [Mfuzz package (Kumar and Futschik 2007) in ‘R’ program]. The “cselection” function was used in Mfuzz to determine cluster number. We used the method proposed by Schwaemmle and Jensen (Schwämmle and Jensen 2010) to estimate the value of fuzziness parameter “m”, which is based on the distribution of distances between genes in a given data set, to set a suitable value for “m”. Maternal genes were classified based on gene expression in both the *Tribolium* ovary and early embryos. Zygotic transcripts and induced zygotic transcripts were identified as mRNAs that were co-regulated in embryo and did not appear in other tested samples. Second, we selected the set of genes whose expression changes greater than or equal to twofold in tissue or embryo in pairwise comparison to other tested samples (edgeR in R program, FDR < 0.01). Edge R software (McCarthy *et al.* 2012) in “R” package was used to normalized the libraries using the trimmed mean of M-values method and for differential gene expression analysis. Genes were kept in the analysis only if the counts per million (CPM) was equal or greater than five in three or more samples.

### Quantitative real-time PCR

The software Primer-3 (http://frodo.wi.mit.edu/) was used to design primers for qPCR analysis. Primers were designed by the rules of highest maximum efficiency, and sensitivity rules were followed to avoid formation of self and hetero-dimers, hairpins and self-complementarity. The primer sequences used in this study are shown in Table S1. In brief, single-strand cDNA was synthesized as follows: 500 ng of total RNA in 11 µl of sterile water previously treated with DNase using the DNA-free kit (Ambion www.ambion.com) following the manufacturer’s instructions. The reverse-transcriptase reaction to generate the cDNA for use in quantitative real-time PCR was carried out using the First Strand cDNA Synthesis kit (Fermentas) as follows: 1 μl of oligo d (T) primer was added to the 11 µl of total RNA. The mixture was heated at 65° for 5 min, and then placed on ice, and the following were added: 4 μl of 5X first-strand buffer, 2 μl of dNTPs, 1 µl of RNase inhibitor, and 1 µl of reverse transcriptase. cDNA synthesis was performed at 42° for 30 min and 50° for 60 min. Reactions were stopped by heating samples at 95° for 2 min.

qRT-PCR was performed in optical 96-well plates on a BioRad Real-Time PCR Detection System using the Absolute QPCR SYBR green Mix (ABgene) to monitor double-stranded DNA synthesis in combination with ROX as a passive reference dye included in the PCR master mix. Amplification conditions were 10 min at 95° to activate the polymerase, followed by 40 cycles at 95° for 30s, 60° for 30s and 72° for 30s. A melting curve analysis was performed in order to verify that the amplicon was at a correct *T_m_* and therefore of the correct length of the predicted transcript. Results were normalized to the mRNA level of *T. castaneum* actin *(*ACT) and ribosomal protein RpSL32 as housekeeping genes (Table S1), and data calculated according to the delta-delta *C*t method. Each experiment was repeated with three independent mRNA samples (biological replicates), and each reaction was repeated three times to minimize intra-experiment variation (technical replicates). All of the results were analyzed with CFX manager software.

### *Drosophila melanogaster* homologs in T. castaneum

The iBeetle web server (http://ibeetle-base.uni-goettingen.de/) (Dönitz *et al.* 2018) was used to identify *T. castaneum* homologs to the *D. melanogaster* genes, which calculated homologs based on OrthoDB. Lists of *D. melanogaster* known maternal and zygotic genes, also genes highly expressed in the *D. melanogaster* germ cells and somatic tissue were retrieved from Table S12-30 of (Arbeitman *et al.* 2002), Table S4, S6, S7 and S8 of (De Renzis *et al.* 2007), additional files 3, 8 and 9 of (Lebo *et al.* 2009) and additional data 21-23 of (Parisi *et al.* 2004). Additional lists of *T. castaneum* genes expressed greater than or equal to twofold change in fertilized and unfertilized eggs were obtained from additional file 1 of (Preuss *et al.* 2012). In this study, we selected the *D. melanogaster* homologs of genes expressed in the *Tribolium* embryo and different tissues, which either showed unique gene expression pattern in clusters by using soft clustering analysis or in pairwise comparisons showed greater than or equal to twofold change in gene expression (FDR <0.01) in edgeR analysis in respective tissues.

### Gene Ontology (GO) and functional annotation

Functional activity annotation was performed on genes in soft clusters and differentially expressed genes greater than or equal to twofold change by using the g:Profiler (g:GOSt Gene Group Functional Profiling) Gene Ontology web server (Reimand *et al.* 2016). To extract GO terms information and enrichment analysis for the selected genes, we used the following parameters, user threshold (p-value) = 0.001. Significance threshold: g: SCS threshold and organism panel was selected as *T. castaneum*.

### Data availability

All raw and processed RNA seq data from the study are available at the gene expression omnibus (GEO) (Edgar *et al.* 2002) database (www.ncbi.nlm.nih.gov/geo), under accession number GSE119739. Supplemental material available at FigShare: https://doi.org/10.25387/g3.7409393.

## Results and Discussion

Here, we report transcriptional profiles during embryo development, male carcass (lacking testis), female carcass (lacking ovary), testis and ovary by deep RNA sequence analysis (Wang *et al.* 2009). We obtained transcriptome reads for 0-5 hr embryos (average from three replicates 33,458,920 reads); 6-11 hr embryos (average from three replicates 29,034,300 reads); male testis (average from two replicates 29,359,190 reads); male carcass (average from three replicates 32,279,190 reads); female ovary (average from three replicates 31,321,690 reads) and female carcass (average from three replicates 32,059,370 reads) as shown in Figure S1. Out of 16,363 total annotated genes, 12,302 were found to have a normalized expression (CPM) greater than or equal to 5 in three or more samples. A Multidimensional Scaling Plot (MDS) of these samples using edgeR (Robinson and Smyth 2008) on the normalized counts showed similarity within sample sets and dissimilarity of gene expression among sample sets, indicating consistency among replicate samples (Figure S2).

**Figure 1:**
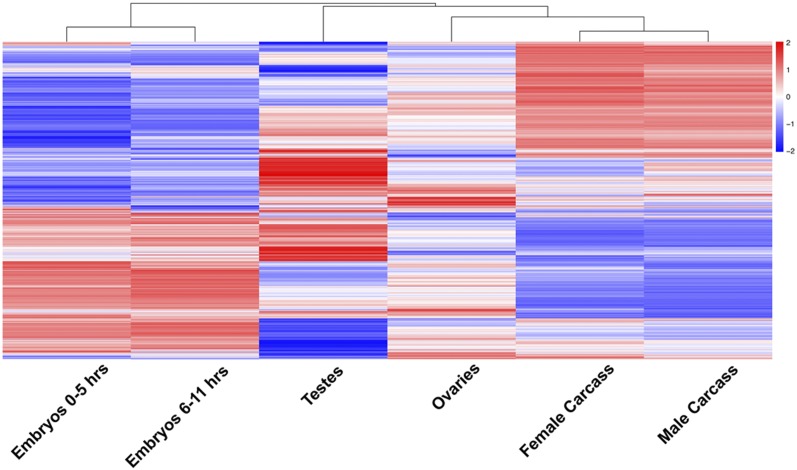
Gene expression pattern revealed by clustering analysis in early embryo and different tissues. Clustering of normalized differentially expressed genes in *Tribolium* based on heat map analysis made using pheatmap package in ‘R’. Euclidean distance was used to estimate pairwise sample distances and sample clustering was done using the k-means algorithm. The columns of the heat map figure indicate samples and the rows represent different genes. The bar color reflects the gene expression levels as TMM normalized log-CPM. Color key indicates the intensity associated with normalized expression values. Red and Blue colors indicate higher expression and lower expression respectively.

To examine the global dynamics of differential gene expression in the *Tribolium* embryo, germ-line and somatic tissues, we compared the normalized expression levels for every sample with every other sample resulting in a global view of gene expression as shown via clustering map (Figure 1). The clustering pattern observed showed that gene expression in male and female germ tissues are substantially different from each other and to both somatic tissues (carcass lacking ovary or testis) and the early embryo. In contrast, the transcriptional profiles of carcass tissues isolated from males and females were highly similar to each other, suggesting minimal sexually dimorphic expression in the adult soma. Similarly, the expression profiles of two embryo stages were similar to each other.

**Figure 2:**
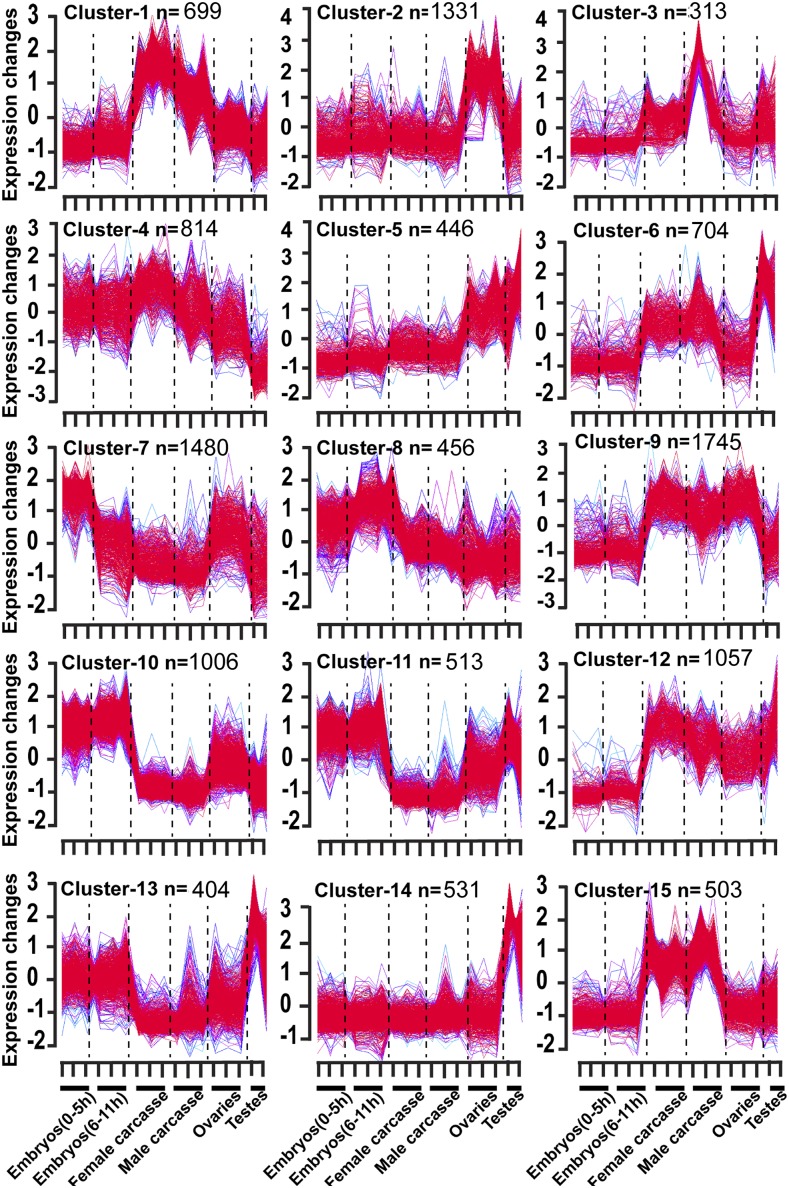
Stage specific changes in gene expression. Genes co-expressed in the *Tribolium* different embryo stages and tested tissues. The X-axis represents samples, samples 1-3 (embryo 0-5 hr), 4-6 (embryo 6-11 hr), 7-9 (female carcass), 10-12 (male carcass), 13-15 (ovary), and 16-17 (testis), with the Y-axis representing change in expression. Line color was assigned to each gene, with red (1) indicating high association in membership to the cluster. Purple, green or blue lines correspond to genes with lower membership values; “n” indicates number of genes assigned to each cluster.

To better understand the major patterns of gene expression, we performed a differential expression analysis between all pairs of samples in the embryo and among tested tissues, classifying individual genes as differentially expressed if they exhibited a twofold change at a false discovery rate of <0.01 (FDR <0.01) as given in Table S2. Additionally, we used a soft clustering algorithm [Mfuzz package (Kumar and Futschik 2007) in ‘R’ program] and established 15 groups of co-regulated genes, with each cluster including between 312 and 1744 expressed genes (Figure 2, Table S3). In the following sections, we present the results from these analyses focusing on each developmental stage/tissue analyzed.

### Identification of maternal gene expression in the Tribolium early embryo

We identified genes differentially expressed in the embryo (0-5 hr) as compared to the ovary, testis, embryo (6-11 hr), and male and female carcasses (Table S2). In pairwise comparisons of embryo (0-5 hr) to other tested samples as given in Table S4, 35 genes were found to be preferentially expressed in the early embryo (0-5 hr). Out of these 35 genes, twelve had homologs of maternal transcripts in the fly embryo (Table S4), including the *TC032769* (caudal) a homolog of the fly gene *(FBgn0000251)*, which is highly expressed in the fly embryo and involved in anterior/posterior patterning formation in the fly embryo. When compared only to the ovary, 62 genes were expressed more than twofold higher in embryo (0-5 hr). Out of these, 20 genes had fly homologs that were also expressed in the fly embryo (Table S4). In strong contrast, comparing gene expression between the early embryo (0-5 hr) to ovary revealed 3639 genes with reduced expression (Table S5). Of these, 1833 genes had a more than fourfold decrease expression in the early embryo (0-5 hr) as compared to ovary, with 27 genes decreased by more than tenfold (Table S5). These genes largely correspond to Cluster-2, which included 1331 genes highly expressed in the ovary (Figure 2), and include those important for the formation and maintenance of this tissue as well as maternally synthesized products required for oocyte formation and early embryogenesis.

**Figure 3:**
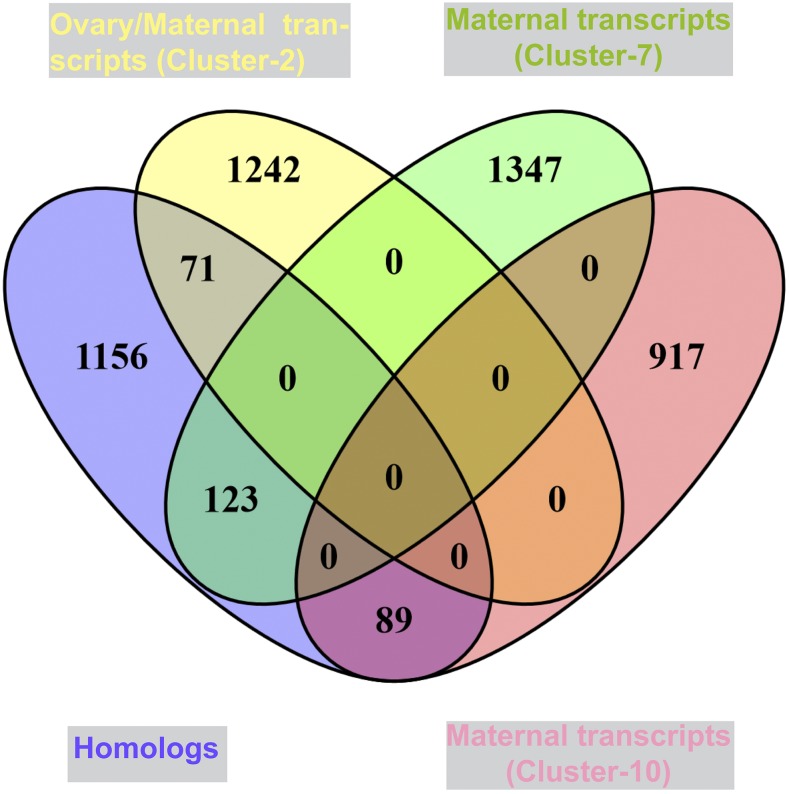
Cluster of highly co-expressed genes in the *Tribolium* ovary and embryo. Genes expressed and grouped together in the *Tribolium* ovary (maternal genes) is shown in Cluster-2 (yellow filled color oval). Genes co-expressed in the *Tribolium* embryo (0-5 hr) and ovary (maternal genes and reduced in expression rapidly) is shown in the Clusters-7 (green filled color oval). Genes co-expressed in both embryo stages and ovary is grouped together in Cluster-10, coral filled color oval (maternal genes). The purple filled color oval represents the known maternal genes. The overlap represents the known maternal genes in *Tribolium*.

As the best studied model system for insect development outside of *Drosophila*, we sought to better identify homologs of maternally deposited *Tribolium* genes that were also maternal in *Drosophila* (Preuss *et al.* 2012; Arbeitman *et al.* 2002) (Figure 3). Among Clusters-2, -7, and -10 we identified 3,817 *Tribolium* genes with expression patterns consistent with those of maternal genes, or 31.02% (maternally deposited) of total transcripts sequenced in this study. In particular, Cluster-7 was assigned genes with higher expression in the *Tribolium* early embryo (0-5 hr) and ovary, but decreased expression after 5 hr (Figure 2). For comparison in *Drosophila*, most maternal genes decrease expression by more than threefold within 6.5 hr of embryonic development or zygotic expression initiation (Arbeitman *et al.* 2002). From prior published studies, we identified 1,439 known maternal genes from *Drosophila* and used these to identify homologs in our dataset, specifically in Cluster-2, 7 and Cluster-10 (Figure 3). Out of 1,439 known *Drosophil*a maternal genes, 71 and 123 homologs of known *Drosophila* maternal genes were identified in Cluster-2 and -7 respectively (Figure 3). For example, *TC002055* present in Cluster-7 is a homolog of *D. melanogaster FBgn0026181*, which is expressed during oogenesis in ovary and deposited to the egg (Verdier *et al.* 2006). *TC015003*, is a homolog of the *Drosophila* FBgn0026206 (*meiotic P26*) (GO:0051321, meiotic cell cycle), which is expressed during oogenesis and was classified as a maternally loaded expressed gene in the fly embryo (Arbeitman *et al.* 2002). Similarly, the *Tribolium intersex* gene (sex determination) had high expression in early embryo (Figure 2, Cluster-7), and the fly homolog of this gene, *FBgn0001276*, is also highly expressed in the fly embryo (Arbeitman *et al.* 2002). Cluster-10 was assigned 1006 genes expressed in both embryo stages and also in the ovary. Of these, 89 were found to have homologs to known maternal genes that persisted during embryogenesis (Figure 3, Cluster-10). For example, *myd88* (*FBgn0033402*) is a known fly maternal gene present in Cluster-10, *myd88* (*TC003185*) is co-expressed in the *Tribolium* early embryo, zygotic stage and ovary.

### Differential expression of genes in the Tribolium zygote

While comparisons between transcript levels in ovary and early embryos can reveal maternally-derived gene products, comparisons with later stage embryos were designed to reveal early zygotic transcripts. At 25°, the embryonic development time of *T. castaneum* is significantly longer than that of *Drosophila*, and the *Tribolium* embryo at less than 5 hr is referred as pre-zygotic (Ninova *et al.* 2016). When we compared post-zygotic embryo (6-11 hr) and pre-zygotic embryo (0-5 hr) samples, 719 genes were identified as early zygotic genes in the *Tribolium* embryos (6-11 hr), with increased expression as compared to early embryo (0-5 hr) (Table S6; Figure S3). This includes gene *TC005375*, whose transcripts were substantially up-regulated in embryo (6-11 hr) compared to all tested samples (Table S6). *TC005375* is a homolog of *D. melanogaster FBgn0002565* (Larval serum protein2) in *Tribolium*. Additionally, expression of *TC015380* (homolog of *D. melanogaster FBgn0033464*) increased more than fourfold in 6-11 hr embryos in comparison 0-5 hr embryos. In the *Tribolium* embryo (6-11 hr), fifteen genes were identified with significantly increased expression in pairwise comparisons with each of the other tested samples (Table S6); out of these, nine had homologs that are early zygotic genes in the fly (Table S6). Genes that are expressed constitutively, particularly in the early embryo, could be potential candidates to use for gene drive systems in insects (Buchman *et al.* 2018).

Cluster-10 (n = 1006 genes) included many genes with high expression in early embryo (0-5 hr), embryo (6-11 hr) and ovary samples. This may represent the persistence of maternal transcripts or, more likely, continued expression during the early zygotic phase.

We identified many genes with expression in both early embryos (0-5 hr) and embryos (6-11 hr) as shown in Figure 2, Cluster-8, which are not expressed in other tested samples. Known zygotic genes were used as queries to identify the any homologs of zygotic expressed genes in *Tribolium*. In cluster analysis, the expression profile pattern of 456 *Tribolium* genes in the embryo samples was similar to that of 137 known zygotic genes in *D. melanogaster* (Figure 2; Cluster-8; Table S6). Five previously characterized early zygotic genes (*TC030997*, *TC008514*, *TC009052*, *TC015380* and *TC033031*) present in Cluster-8 were significantly up-regulated in the *Tribolium* embryos (6-11 hr).

### Identification of genes highly expressed in the Tribolium male germ-line

**Figure 4:**
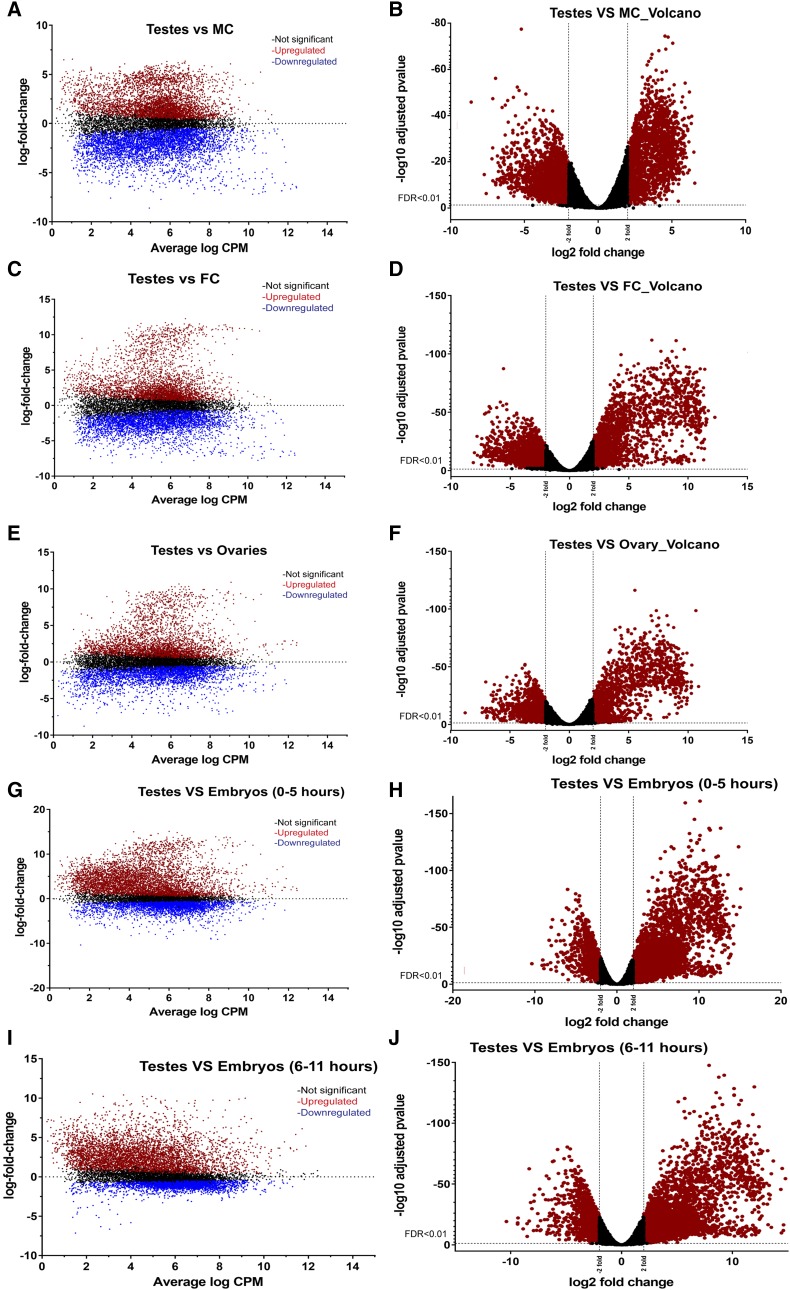
Gene expression patterns in the *Tribolium* testis. Differential genes expression in the *Tribolium* testis compared to male carcass (A –B), female carcass (C-D) ovary (E-F), embryo (0-5 hr) (G-H) and embryo (6-11 hr) (I-J). Colored dots represent individual differentially expressed genes. Red and blue color dots indicate significantly differentially expressed genes; red colored dots on Volcano plots are significant and above twofold or below twofold. N= number of genes DE in greater than or equal to twofold change or lesser than or equal to twofold change.

**Figure 5:**
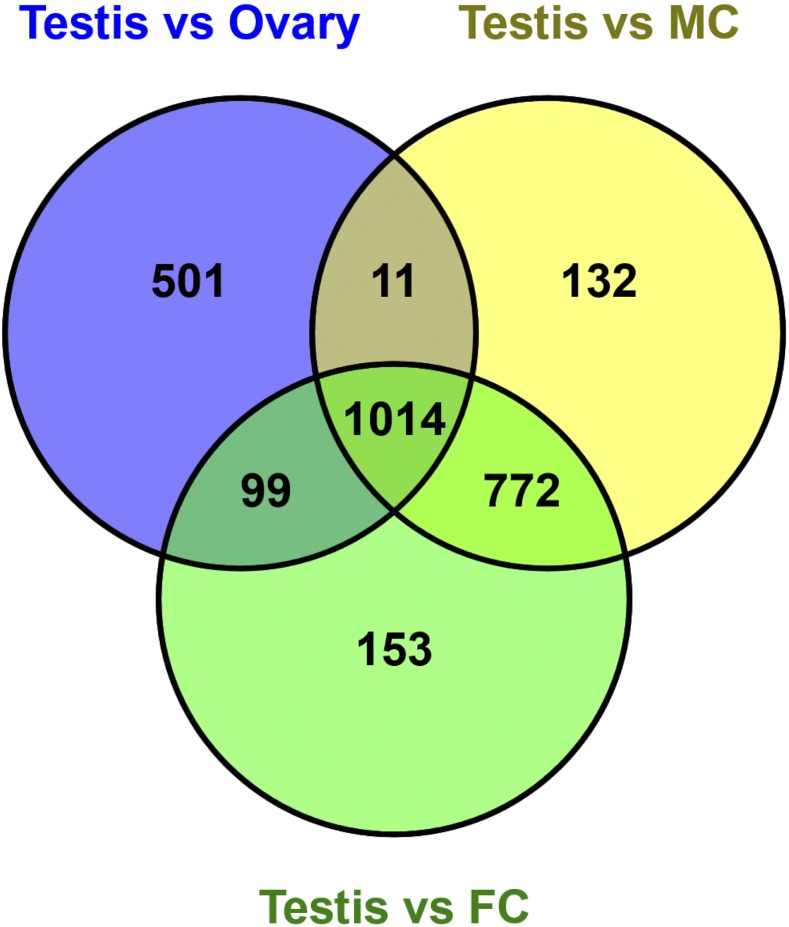
Differential increased gene expression in the *Tribolium* testis. Venn diagrams showing highly expressed genes in the *Tribolium* testis compared to ovary, male and female carcasses.

Cluster-14 was assigned 531 genes, with expression observed primarily in the testis (Figure 2, Table S3). Pairwise comparisons with other samples (Figure 4A-J) revealed 1,014 genes with increased expression in the testis when compared to the ovary, female and male carcasses (Figure 5, Table S7). Genes highly expressed in the *Tribolium* testis were examined for homologs from *D. melanogaster*, leading to the identification of 272 genes with orthologs known to be expressed preferentially in the testis- of the fly (Table S7). Differential high expression of genes in the testis may play specific roles in spermatogenesis and development. For example, the gene *TC032055* had the highest expression (greater than tenfold change) in testis as compared to the other samples as shown in Table S7. This gene is a homolog of *D. melanogaster FBgn0036093* that has a domain of unknown function. *FBgn0036093* has two transcripts and two different polypeptides and has expression observed during the pupal period and in adult males. The *Tribolium* homolog has two transcripts (XM_008203121.2 and XR_001575917.1).

Similarly, another gene highly expressed in the testis was *TC006703*, which codes for the DNA double-strand break repair Rad50 ATPase. Transcript abundance for this gene ranges from a peak of moderately high expression in testis to very low expression in female carcass (Table S7). Rad50 ATPase is an essential component of MRN (Mre11-Rad50-Nbs1) complex; its molecular function is involved in 3′-5′ exonuclease activity and in biological process double-strand break repair via homologous recombination. The MRN complex contributes to processes such as recombination, DNA damage repair, genome stability and meiosis in germ cells. In *Drosophila*, Rad50 also forms a complex with Mre11 to cap telomeres during embryogenesis. Its abundance in the *Tribolium* testis may signify a special need for homology-based DNA repair during spermatogenesis.

Eleven Tubulin family members from the genome of *T. castaneum* were previously described; these were characterized as alpha tubulins, beta tubulins, gamma tubulins, delta tubulins and epsilon tubulins (Siebert *et al.* 2008). *β_2_-tubulin* specific *cis*-elements from different insects such as *D. melanogaste*r, *B. mori*, *A. aegypti* and *A. gambiae* have been used to drive transgene expression in the testis (Michiels *et al.* 1989; Xu *et al.* 2015; Smith *et al.* 2007; Catteruccia *et al.* 2005). We identified four different β-tubulin homologs *TC009589*, *TC034766*, *TC010829*, and *TC009035* from *T. castaneum* reference genome (Figure S4A). The genetic structure of these four β-tubulins homologs from *T. castaneum* is described in the Figure S4B. We found that only *TC009035* was highly expressed (ninefold over other tissues) in testis (Table S7). *TC009035* is a homolog of *D. melanogaster* β-Tub85D (*CG9359*) and *CG9222* (*FBgn0031784*), with the β-Tub85D gene highly expressed in the *D. melanogaster* testis (Graveley *et al.* 2011). In *Drosophila*, this gene is transcribed in late third larval instar before the onset of meiosis in the developing testis and remains active throughout adulthood (Kemphues *et al.* 1982). A lower level of expression for β-Tub85D was also reported in other tissues such as in adult carcass and larval fat body (Gelbart and Emmert 2013).

**Figure 6:**
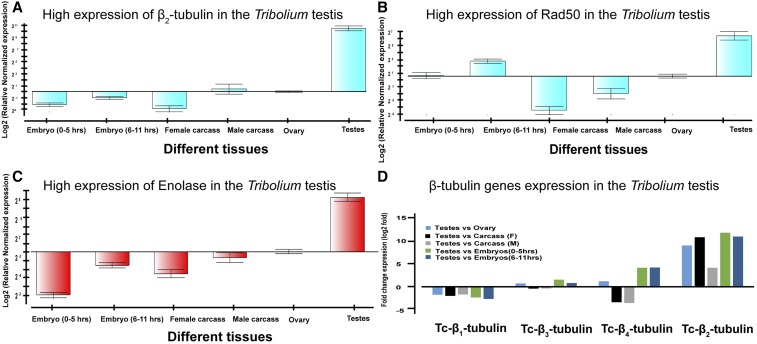
Quantitative real time PCR based validation of highly expressed genes in the testes. Normalized expression of *β_2_-tubulin* (A), *Rad50* (B) and *Enolase* (C) was determined in testis tissue compared to ovary, male carcass, female carcass, embryo (0-5 hr) and embryo (6-11 hr) based on qPCR analysis. Fold change expression of four *β-tubulins* (*Tc-β_1_*, *Tc-β_2_*, *Tc-β_3_* and *Tc-β_4_ tubulin*) in testis compared to ovary, male carcass, female carcass, embryo (0-5 hr) and embryo (6-11 hr) based on RNA seq analysis (D).

To validate RNAseq data for genes expressed predominantly in the testis, we performed qPCR analysis as an independent measure of gene expression levels for *TcRad50*, *Tc-β_2_-tubulin* and an *enolase* gene. In each case, the qPCR data analysis demonstrated robust expression in the *Tribolium* testis (Figure 6 A-D), confirming our RNAseq data and analysis.

### Differential gene expression in the Tribolium female germ-line

**Figure 7:**
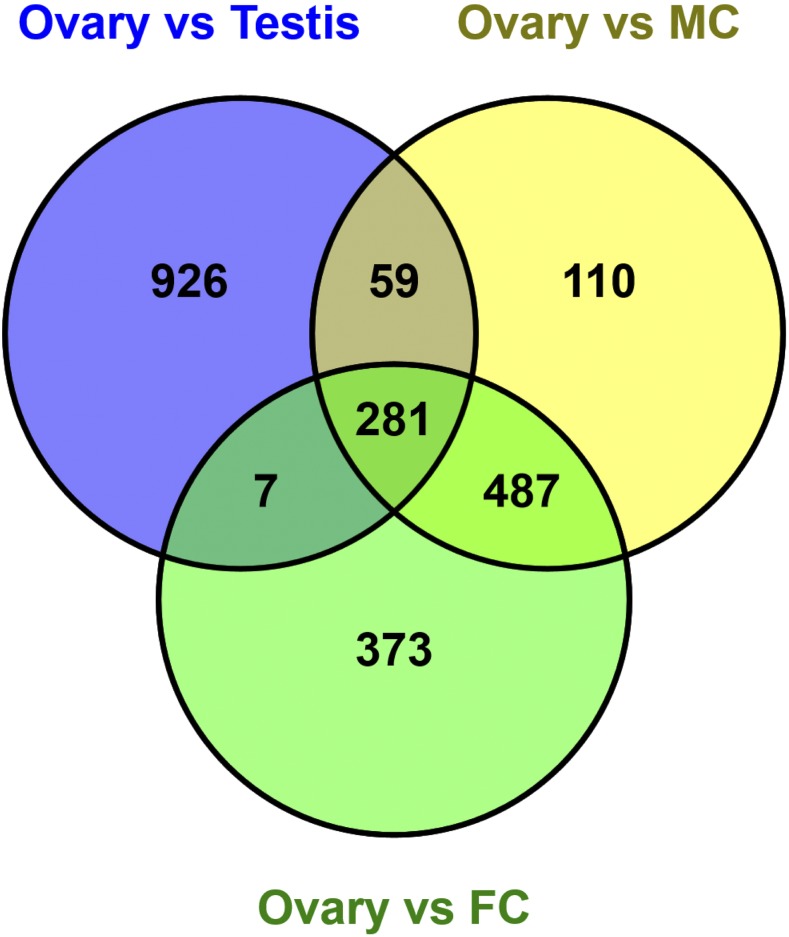
Highly expressed genes in the *Tribolium* ovary tissue. Venn diagrams representing increased gene expression greater than or equal to twofold in the *Tribolium* ovary in these comparisons (*i.e.*, “Ovary vs Testis”, “Ovary vs Male carcass” and “Ovary vs Female carcass”).

As stated above, the analysis of genes assigned to Cluster-2 revealed a group of genes that are transcribed in ovary and deposited into embryo but rapidly reduced in expression in embryo. Cluster-2 was assigned 1331 genes, out of which 71 were *Tribolium* homologs of fly female-biased genes, which were transcribed only in the ovary and may have a role in ovary tissue development and oocyte synthesis. Two genes out of these 71 genes were *TC009818* (homolog of *D. melanogaster FBgn0020278*; *loco*) and *TC013108* (homolog of *D. melanogaster FBgn0003187*; *quail*) which have a role in oocyte dorsal/ventral axis specification and ovarian nurse cell to oocyte transport respectively. *D. melanogaster loco* may be required for nurse cell dumping during oogenesis (Pathirana *et al.* 2001). Mutations in *D. melanogaster quail* result in female sterility (Mahajan-Miklos and Cooley 1994). Our data suggests that the homologs of these two genes may be needed for egg production during oogenesis (Figure 2, Cluster-2). As an alternative method to identify differentially expressed genes in ovary, we compared gene expression profiles in ovary to all tested samples (Figure S5A-H), considering a threshold of greater than or equal to twofold change in gene expression in ovary as compared to testis, female and male carcasses at an FDR value < 0.01 (Table S8). By this method, 281 genes were identified as differentially expressed in the ovary tissue as compared to all other samples (Figure 7; Table S8). Such genes are good candidates for donating *cis*-acting control elements. For example, *TC034053*, which has greater than threefold (q-value 8.99x10^−18^) increased expression in the *Tribolium* ovary compared to testis, is a homolog of the *D. melanogaster* gene *FBgn0039972* (meiosis arrest female protein 1), which has known expression in adult female and early embryo (Table S8).

### Identification of genes differentially expressed in the Tribolium germ-line of either sex

**Figure 8:**
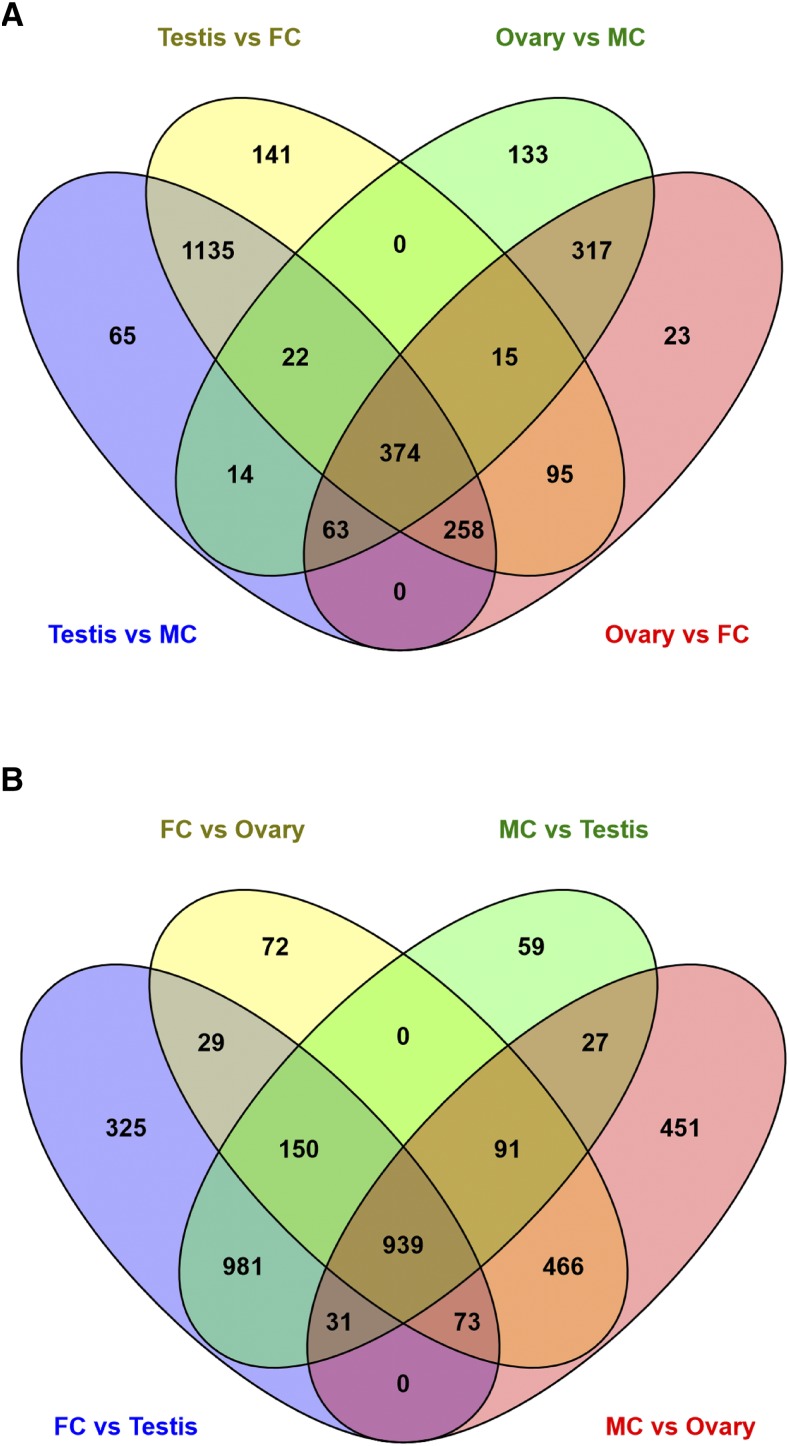
Differential gene expression in the *Tribolium* germ-line. List of genes with significantly increased expression in germ-line tissues. Genes in the common overlapping set show increased genes expression in germ-line such as in testis, ovary or both (A). Genes with significantly decreased expression in germ-line tissues as compared to the rest of the body (B).

Sexual reproduction involves specialized variation of certain cellular processes in germ-line tissues such as sperm and egg production. To identify genes differentially regulated in the *Tribolium* germ-line, we compared expression levels of each expressed gene in the male germ-line tissues (testis) to expression in both male carcass and female carcass; simultaneously we compared gene expression levels in the ovary to expression levels in both male and females carcasses (Figure 8A). From these comparisons, 374 genes had higher expression in the germ-line greater than or equal to twofold in both sexes relative to somatic tissues (Figure 8A; Table S9). Of these germ-line expressed genes, 81 had homologs in *Drosophila* that are also preferentially expressed in the germ-line (Table S9). For example, *TC001383* (fourfold change; q-value 2.91x 10^−23^) is a homolog of a *D. melanogaster* gene (*FBgn0001086*), which is involved in the anaphase promoting complex in mitosis that functions to regulate the three mitotic cyclins A, B and B3 in the egg and drive anaphase progression during meiosis (Frise *et al.* 2010).

From the same comparisons, we also sought to identify genes with significantly decreased expression in germ-line tissues as compared to the rest of the body (Figure 8B, Table S10). We identified 939 genes, with significantly less expression in the male and female germ-line as compared to the dissected carcasses without these organs; with 27 genes more highly expressed only in the male carcass and 29 genes expressed preferentially in the female carcass (Figure 8B and Table S10). Genes expressed preferentially in *Tribolium* somatic tissues were compared to their *D. melanogaster* homologs, leading to the identification of 264 homologs from *D. melanogaster* with corresponding soma-biased genes in *Tribolium* (Table S10). Among these, the expressions of 10 *D. melanogaster* homologs were also male-biased and four *D. melanogaster* homologs were female biased (Table S10). For example, *TC000087* is a homolog of the *FBgn0001208* (*henna*), which has a role in the biosynthesis of pteridin eye pigment. Similarly, *TC010476* (greater than sixfold change; q-value 4.59X10^−14^) is a homolog of the *D. melanogaster* gene *FBgn0005633* (*flightin*). Flightin (Fln) a flight muscle specific protein in *D. melanogaster*, and is not expressed in other muscle types and is involved for the correct assembly of the myosin thick filament. Clusters-1 and -15 include genes with expression in adult somatic tissues, while Cluster-3 (313 genes) had the smallest number of genes of all the clusters (Figure 2, Table S3) and was assigned genes with high expression in male carcass (lacking testis). Finally, Cluster-12 included 1057 genes (Figure 2, Table S3), which were highly expressed in tissues from the adult stages analyzed, including both somatic and germ-line tissues.

### Analysis of gene function through homology

The function of each gene was predicted based on the annotation categories assigned and their enrichment score in specific gene expression patterns observed in the beetle genome. This assignment allowed for the identification of over-represented gene functions present in each cluster using g: Profiler (*P* < 0.001) (Reimand *et al.* 2016) web server (Table S11 and S12).

Genes with functions such as cytoskeleton activity in cellular components (p-value = 7.8x10^−8^) and microtubule-based processes were enriched in Cluster-7 (Table S11). Genes associated with GO terms like transcription factor activity and binding activity as well as those that code for ubiquitin proteins, nucleus, nucleic acid metabolic process, DNA replication and microtubule motor activity proteins etc., were enriched in Cluster-10 (Table S11). Integral component of membrane was the most abundant cellular component category found in the ovary-expressed genes examined while in the biological process category; the majority of these genes were categorized as cell adhesion proteins (Table S11, Cluster-2). In the molecular function group, genes in Cluster-2 were enriched for ion binding and transporter proteins (Table S11).

Functional categories overrepresented in both germ-line and somatic tissues of male and female carcasses are presented in Table S12. In testis, enriched functional categories include microtubule-based processes; microtubule associated complex activities and movement of cell or subcellular component like biological process (Table S12). While the categories found highly enriched in genes present in both germ-cells include cell cycle (p-value = 2.1x10^−10^) and cell division (p-value = 2.9x10^−4^) (Table S12), genes with catalytic activity, metabolic process, hydrolase activity, binding activity, chitin and peptidases functions were found highly over-represented in soma-biased expressed genes (Table S12).

### CONCLUSIONS

Important genetic and genomic resources, including a high quality genome sequence (Tribolium Genome Sequencing Consortium *et al.* 2008), have contributed to the ability to perform transcriptomic studies in *Tribolium* (Ninova *et al.* 2016; Preuss *et al.* 2012; Park *et al.* 2008; Altincicek *et al.* 2013), progressively making *Tribolium* a preferred model organism for molecular biology studies. In the present study, we have reported on the deep transcriptome of the *Tribolium* pre-zygotic embryo, zygotic embryo, testis, ovary, female adult soma, and male adult soma, and present the transcriptional profiles for three quarters (∼75%) of all predicted *Tribolium* genes in these tissues/stages (Tribolium Genome Sequencing Consortium *et al.* 2008).

Metazoan embryonic development is coordinated by both maternal RNAs deposited into the egg and the transcription of zygotic genes after fertilization. Depending on the insect species maternal transcripts persist a specific amount of time (Arbeitman *et al.* 2002). In *Tribolium* for example, we observed one set of maternal genes whose transcripts level substantially decreased after >6 hr following fertilization.

We observed the similarity and dissimilarity for maternally deposited homolog genes and early zygotic homologs in both *Drosophila* and *Tribolium*. The study of regulatory elements required for proper gene expression is crucial for understanding the molecular basis of evolution. Rather than the acquisition of new genes or modification of gene products, it is changes in gene regulatory elements that are often responsible for the evolution of new morphology (Carroll 2008). Wolff *et al.* (1998) studied both evolutionary conserved and diverged enhancers in *Tribolium* and *Drosophila* and reported that a major shift in the evolutionary transition from short (*Tribolium*) to long germ (*Drosophila*) embryogenesis is due to regulatory adaptation during the evolution of developmental processes (Wolff *et al.* 1998).

Gene expression levels are tightly regulated in ovary, testis, carcasses and early embryo and are likely controlled by cis elements that could be used to drive the expression of transgenic constructs for embryogenesis studies and genetic pest management strategies (Hammond *et al.* 2016; Buchman *et al.* 2018; Lai *et al.* 2018). Cis elements associated with genes identified in this study could be evaluated for functional activity using established reporter assays in *Tribolium* (Lai *et al.* 2018). Thus, the data we describe here can be mined for elements to construct synthetically engineered gene drive systems in *Tribolium* and to some extent other Coleopteran pest insects as well. Such gene drive systems have potential to replace, alter, or suppress wild populations of significant crop pests or other economical important insects (Buchman *et al.* 2018; Akbari *et al.* 2014). For example, recent strategies have featured the creation of male insects whose sperm express a transgene-based toxin that suppresses the insect population (Klein *et al.* 2012).

## References

[bib1] Akbari O. S. Antoshechkin I. Amrhein H. Williams B. Diloreto R., 2013 The Developmental Transcriptome of the Mosquito Aedes aegypti, an Invasive Species and Major Arbovirus Vector. G3 (Bethesda) 3: 1493–1509. 10.1534/g3.113.006742 23833213 PMC3755910

[bib2] Akbari O. S. Chen C. H. Marshall J. M. Huang H. Antoshechkin I., 2014 Novel synthetic Medea selfish genetic elements drive population replacement in *Drosophila*; a theoretical exploration of Medea-dependent population suppression. ACS Synth. Biol. 3: 915–928. 10.1021/sb300079h 23654248 PMC3742681

[bib3] Altincicek B. Elashry A. Guz N. Grundler F. M. W. Vilcinskas A., 2013 Next Generation Sequencing Based Transcriptome Analysis of Septic-Injury Responsive Genes in the Beetle *Tribolium castaneum*. PLoS One 8: e52004 10.1371/journal.pone.0052004 23326321 PMC3541394

[bib4] Arbeitman M. N. Furlong E. E. M. Imam F. Johnson E. Null B. H., 2002 Gene expression during the life cycle of *Drosophila melanogaster*. Science 279: 2270–2275 (erratum: Science 298: 1172). 10.1126/science.1072152 12351791

[bib5] Buchman A. Marshall J. M Ostrovski D. Yang T. Akbari O. S., 2018 Synthetically Engineered Medea Gene Drive System in the Worldwide Crop Pest, *D. suzukii*. Proc. Natl. Acad. Sci. USA 115: 4725–4730. 10.1073/pnas.1713139115 29666236 PMC5939061

[bib6] Carroll S. B., 2008 Evo-devo and an expanding evolutionary synthesis: A genetic theory of morphological evolution. Cell 134: 25–36. 10.1016/j.cell.2008.06.030 18614008

[bib7] Catteruccia F. Benton J. P. Crisanti A., 2005 An Anopheles transgenic sexing strain for vector control. Nat. Biotechnol. 23: 1414–1417. 10.1038/nbt1152 16244659

[bib8] De Renzis S. Elemento O. Tavazoie S. Wieschaus E. F., 2007 Unmasking activation of the zygotic genome using chromosomal deletions in the *Drosophila* embryo. PLoS Biol. 5: e117 (erratum: PLoS Biol. 5: 10.1371/journal.pbio.0050117). 10.1371/journal.pbio.0050213 17456005 PMC1854917

[bib9] Dönitz J. G. Gerischer L. Hahnke S. Pfeiffer S. Bucher G., 2018 Expanded and updated data and a query pipeline for iBeetle-Base. Nucleic Acids Res. 46: D831–D835. 10.1093/nar/gkx984 29069517 PMC5753255

[bib10] Edgar R. Domrachev M. Lash A. E., 2002 Gene Expression Omnibus: NCBI gene expression and hybridization array data repository. Nucleic Acids Res. 30: 207–210. 10.1093/nar/30.1.207 11752295 PMC99122

[bib11] Frise E. Hammonds A. S. Celniker S. E., 2010 Systematic image-driven analysis of the spatial *Drosophila* embryonic expression landscape. Mol. Syst. Biol. 6: 345 10.1038/msb.2009.102 20087342 PMC2824522

[bib12] Gelbart, W. M., and D. B. Emmert, 2013 FlyBase High Throughput Expression Pattern Data, FBrf0221009, FlyBase analysis.

[bib13] Graveley B. R. Brooks A. N. Carlson J., 2011 The developmental transcriptome of *Drosophila melanogaster*. Nature 471: 473–479. 10.1038/nature09715 21179090 PMC3075879

[bib14] Guo X. H. Li M. Gao P. F. Cao G. Q. Cheng Z. M., 2018 Novel splice isoforms of pig myoneurin and their diverse mRNA expression patterns. Asian-Australas. J. Anim. Sci. 31: 1581–1590. 10.5713/ajas.17.0911 29747493 PMC6127594

[bib15] Hale M. C. Xu P. Scardina J. Wheeler P. A. Thorgaard G. H., 2011 Differential gene expression in male and female rainbow trout embryos prior to the onset of gross morphological differentiation of the gonads. BMC Genomics 12: 404 10.1186/1471-2164-12-404 21824436 PMC3166948

[bib16] Hammond A. Galizi R. Kyrou K. Simoni A. Siniscalchi C., 2016 A CRISPR-Cas9 gene drive system-targeting female reproduction in the malaria mosquito vector *Anopheles gambiae*. Nat. Biotechnol. 34: 78–83. 10.1038/nbt.3439 26641531 PMC4913862

[bib17] Hunt T. Bergsten J. Levkanicova Z. Papadopoulou A. John O. S., 2007 A comprehensive phylogeny of beetles reveals the evolutionary origins of a superradiation. Science 318: 1913–1916. 10.1126/science.1146954 18096805

[bib18] Jiang Z. F. Machado C. A., 2009 Evolution of sex-dependent gene expression in three recently diverged species of *Drosophila*. Genetics 183: 1175–1185. 10.1534/genetics.109.105775 19720861 PMC2778969

[bib19] Kemphues K. J. Kaufman T. C. Raff R. A. Raff E. C., 1982 The testis-specific beta-tubulin subunit in *Drosophila melanogaster* has multiple functions in spermatogenesis. Cell 31: 655–670. 10.1016/0092-8674(82)90321-X 6819086

[bib20] Kim D. Landmead B. Salzberg S. L., 2015 HISAT: a fast spliced aligner with low memory requirements. Nat. Methods 12: 357–360. 10.1038/nmeth.3317 25751142 PMC4655817

[bib21] Klein T. A. N. Windbichler A. Deredec A. B. Benedict M. Q., 2012 Infertility resulting from transgenic I-PpoI male *Anopheles gambiae* in large cage trials. Pathog. Glob. Health 106: 20–31. 10.1179/2047773212Y.0000000003 22595271 PMC4001508

[bib22] Kumar L. Futschik M. E., 2007 Mfuzz: a software package for soft clustering of microarray data. Bioinformation 2: 5–7. 10.6026/97320630002005 18084642 PMC2139991

[bib23] Lai Y. T. Deem K. D. Borras-Castells F. Sambrani N. Rudolf H., 2018 Enhancer identification and activity evaluation in the red flour beetle, Tribolium castaneum. Development 145: dev160663. 10.1242/dev.160663 PMC11736658 29540499

[bib24] Lebo M. S. Sanders L. E. Sun F. Z. Arbeitman M. N., 2009 Somatic, germline and sex hierarchy regulated gene expression during *Drosophila* metamorphosis. BMC Genomics 10: 80 10.1186/1471-2164-10-80 19216785 PMC2656526

[bib25] Mahajan-Miklos S. Cooley L., 1994 The Vilin-like protein encoded by the *Drosophila* quail gene is required for actin bundle assembly during oogenesis. Cell 78: 291–301. 10.1016/0092-8674(94)90298-4 8044841

[bib26] Matson C. K. Zarkower D., 2012 Sex and the singular DM domain: insights into sexual regulation, evolution and plasticity. Nat. Rev. Genet. 13: 163–174. 10.1038/nrg3161 22310892 PMC3595575

[bib27] McCarthy D. J. Chen Y. Smyth G. K., 2012 Differential expression analysis of multifactor RNA-Seq experiments with respect to biological variation. Nucleic Acids Res. 40: 4288–4297. 10.1093/nar/gks042 22287627 PMC3378882

[bib28] McIntyre L. M. Bono L. M. Genissel A. Westerman R. Junk D., 2006 Sex-specific expression of alternative transcripts in *Drosophila*. Genome Biol. 7: R79 10.1186/gb-2006-7-8-r79 16934145 PMC1779584

[bib29] Michiels F. Gasch A. Kaltschmidt B. Renkawitzpohl R., 1989 A 14-BP promoter element directs the testis specificity of the *Drosophila*-Beta-2 tubulin gene. EMBO J. 8: 1559–1565. 10.1002/j.1460-2075.1989.tb03540.x 2504583 PMC400987

[bib30] Ninova M. Ronshaugen M. Griffiths-Jones S., 2016 MicroRNA evolution, expression, and function during short germband development in *Tribolium castaneum*. Genome Res. 26: 85–96. 10.1101/gr.193367.115 26518483 PMC4691753

[bib31] Papathanos P. A. Bossin H. C. Benedict M. Q. Catteruccia F. M. C. A. Alphey L., 2009 Sex separation strategies: past experience and new approaches. Malar. J. 8: S5 10.1186/1475-2875-8-S2-S5 PMC2777327 19917075

[bib32] Parisi M. Nuttall R. Edwards P. Minor J. Naiman D., 2004 A survey of ovary-, testis-, and soma-biased gene expression in *Drosophila melanogaster* adults. Genome Biol. 5: R40 10.1186/gb-2004-5-6-r40 15186491 PMC463073

[bib33] Park Y. Aikins J. Wang L. J. Beeman R. W. Oppert B., 2008 Analysis of transcriptome data in the red flour beetle, Tribolium castaneum. Insect Biochem. Mol. Biol. 38: 380–386. 10.1016/j.ibmb.2007.09.008 18342244 PMC2387101

[bib34] Pathirana S. Zhao D. Bownes M., 2001 The *Drosophila* RGS protein Loco is required for dorsal/ventral axis formation of the egg and embryo, and nurse cell dumping. Mech. Dev. 109: 137–150. 10.1016/S0925-4773(01)00557-3 11731228

[bib35] Pokorná M. Kratochvíl L., 2009 Phylogeny of sex-determining mechanisms in squamate reptiles: are sex chromosomes an evolutionary trap? Zool. J. Linn. Soc. 156: 168–183. 10.1111/j.1096-3642.2008.00481.x

[bib36] Preuss K. M. Lopez J. A. Colbourne J. K. Wade M. J., 2012 Identification of maternally-loaded RNA transcripts in unfertilized eggs of *Tribolium castaneum*. BMC Genomics 13: 671 10.1186/1471-2164-13-671 23181844 PMC3536564

[bib37] Prince E. G. Kirkland D. Demuth J. P., 2010 Hyperexpression of the X Chromosome in Both Sexes Results in Extensive Female Bias of X–Linked Genes in the Flour Beetle. Genome Biol. Evol. 2: 336–346. 10.1093/gbe/evq024 20624738 PMC2942036

[bib38] Reimand J. T. Arak P. Adler L. Kolberg S. Reisberg H., 2016 a web server for functional interpretation of gene lists. Nucleic Acids Res. 44: W83–W89. 10.1093/nar/gkw199 27098042 PMC4987867

[bib39] Rinn J. L. Snyder M., 2005 Sexual dimorphism in mammalian gene expression. Trends Genet. 21: 298–305. 10.1016/j.tig.2005.03.005 15851067

[bib40] Robinson M. D. Smyth G. K., 2008 Small-sample estimation of negative binomial dispersion, with applications to SAGE data. Biostatistics 9: 321–332. 10.1093/biostatistics/kxm030 17728317

[bib41] Ruan J. Guo F. Wang Y. Y. Li X. G. Wan S. B., 2018 Transcriptome analysis of alternative splicing in peanut (Arachis hypogaea L.). BMC Plant Biol. 18: 139 10.1186/s12870-018-1339 29973157 PMC6032549

[bib42] Salz H. K., 2011 Sex determination in insects: a binary decision based on alternative splicing. Curr. Opin. Genet. Dev. 21: 395–400. 10.1016/j.gde.2011.03.001 21474300 PMC3134629

[bib43] Schutt C. Nothiger R., 2000 Structure, function and evolution of sex-determining systems in Dipteran insects. Development 127: 667–677. 10648226 10.1242/dev.127.4.667

[bib44] Schwämmle V. Jensen O. N., 2010 A simple and fast method to determine the parameters for fuzzy c-means cluster analysis. Bioinformatics 26: 2841–2848. 10.1093/bioinformatics/btq534 20880957

[bib45] Siebert K. S. Lorenzen M. D. Brown S. J. Park Y. Beeman R. W., 2008 Tubulin superfamily genes in Tribolium castaneum and the use of a Tubulin promoter to drive transgene expression. Insect Biochem. Mol. Biol. 38: 749–755. 10.1016/j.ibmb.2008.04.007 18625397

[bib46] Smith R. C. Walter M. F. Hice R. H. O’Brochta D. A. Atkinson P. W., 2007 Testis-specific expression of the beta 2 tubulin promoter of Aedes aegypti and its application as a genetic sex-separation marker. Insect Mol. Biol. 16: 61–71. 10.1111/j.1365-2583.2006.00701.x 17257209

[bib47] Thoemke K. Yi W. S. Ross J. M. Kim S. Reinke V., 2005 Genome-wide analysis of sex-enriched gene expression during *C. elegans* larval development. Dev. Biol. 284: 500–508. 10.1016/j.ydbio.2005.05.017 15987632

[bib48] Tribolium Genome Sequencing Consortium; Richards S. G. Gibbs R. A. Weinstock G. M. Brown S. J. Denell R., 2008 The genome of the model beetle and pest Tribolium castaneum. Nature 452: 949–955. 10.1038/nature06784 18362917

[bib49] Verdier V. Johndrow J. E. Betson M. Chen G. C. Hughes D. A., 2006 Drosophila Rho-kinase (DRok) is required for tissue morphogenesis in diverse compartments of the egg chamber during oogenesis. Dev. Biol. 297: 417–432. 10.1016/j.ydbio.2006.05.016 16887114 PMC2504748

[bib50] Verhulst E. C. Van de Zande L. Beukeboom L. W., 2010 Insect sex determination: it all evolves around transformer. Curr. Opin. Genet. Dev. 20: 376–383. 10.1016/j.gde.2010.05.001 20570131

[bib51] Viets B. E. Ewert M. A. Talent L. G. Nelson C. E., 1994 Sex determining mechanisms in squamate reptiles. J. Exp. Zool. 270: 45–56. 10.1002/jez.1402700106

[bib52] Wang Z. Gerstein M. Snyder M., 2009 RNA-Seq: a revolutionary tool for transcriptomics. Nat. Rev. Genet. 10: 57–63. 10.1038/nrg2484 19015660 PMC2949280

[bib53] West S. A. Reece S. E. Sheldon B. C., 2002 Sex ratios. Heredity 88: 117–124. 10.1038/sj.hdy.6800018 11932770

[bib54] Wolff C. Schroder R. Schulz C. Tautz D. Klingler M., 1998 Regulation of the Tribolium homologues of caudal and hunchback in Drosophila: evidence for maternal gradient systems in a short germ embryo. Development 125: 3645–3654. 9716530 10.1242/dev.125.18.3645

[bib55] Xu J. Bi H. Chen R. Aslam A. F. M. Li Z., 2015 Transgenic characterization of two testis-specific promoters in the silkworm, Bombyx mori. Insect Mol. Biol. 24: 183–190. 10.1111/imb.12144 25387604

[bib56] Zhang Y. Sturgill D. Parisi M. Kumar S. Oliver B., 2007 Constraint and turnover in sex-biased gene expression in the genus Drosophila. Nature 450: 233–237. 10.1038/nature06323 17994089 PMC2386141

